# Ob/Gyn resident self-perceived preparedness for minimally invasive surgery

**DOI:** 10.1186/s12909-020-02090-9

**Published:** 2020-06-05

**Authors:** Jordan S. Klebanoff, Cherie Q. Marfori, Maria V. Vargas, Richard L. Amdur, Catherine Z. Wu, Gaby N. Moawad

**Affiliations:** 1 Department of Obstetrics and Gynecology, The George Washington University School of Medicine and Health Sciences, Washington, D.C, USA; 2 Department of Surgery, The George Washington University School of Medicine and Health Sciences, Washington, D.C, USA

**Keywords:** Gynecology, Minimally invasive gynecologic surgery, Surgery, Surgical education, Residency

## Abstract

**Background:**

Very little is known regarding the readiness of senior U.S. Ob/Gyn residents to perform minimally invasive surgery. This study aims to evaluate the self-perceived readiness of senior Ob/Gyn residents to perform complex minimally invasive gynecologic surgery as well as their perceptions of the minimally invasive gynecologic surgery subspecialty.

**Methods:**

We performed a national survey study of 3rd and 4th year Ob/Gyn residents. A novel 58-item survey was developed and sent to residency program directors and coordinators with the request to forward the survey link along to their senior residents.

**Results:**

We received 158 survey responses with 84 (53.2%) responses coming from 4th year residents and 74 (46.8%) responses from 3rd year residents. Residents who train with graduates of a fellowship in minimally invasive gynecologic surgery felt significantly more prepared to perform minimally invasive surgery compared to residents without this exposure in their training. The majority of senior residents (71.5%) feel their residency training adequately prepared them to be a competent minimally invasive gynecologic surgeon. However, only 50% feel prepared to perform a laparoscopic hysterectomy on a uterus greater than 12 weeks size, 29% feel prepared to offer a vaginal hysterectomy on a uterus 12-week size or greater, 17% feel comfortable performing a laparoscopic myomectomy, and 12% feel prepared to offer a laparoscopic hysterectomy for a uterus above the umbilicus.

**Conclusions:**

The majority of senior U.S. Ob/Gyn residents feel prepared to provide minimally invasive surgery for complex gynecologic cases. However, surgical confidence in specific procedures decreases when surgical complexity increases.

## Highlights

- The majority of senior US Ob/Gyn residents have self perceived preparedness to perform minimally invasive surgery
- As surgical procedures become increasingly complex resident self perceived preparedness to perform these procedures decreases
- Senior Ob/Gyn residents are least confident in performing vaginal hysterectomies requiring vaginal morcellation for tissue extraction, laparoscopic hysterectomies when the uterus is above the umbilicus, excision of stage 3 or 4 endometriosis, laparoscopic myomectomies requiring suturing, and laparoscopic myomectomies requiring suturing of more than one hysterotomy
- Residents who train with graduates of a fellowship in MIGS felt significantly more prepared to perform MIGS compared to residents without this exposure in their training

## Background

There is a growing concern that the traditional 4-year Ob/Gyn residency does not allow enough time for comprehensive surgical training. Restricted resident duty hours and increased medical management of gynecologic conditions result in lower surgical caseloads among graduates [1, 2]. A national conversation of whether or not “tracking,” which allows for increased clinical time in specific areas of interest based on resident preference, should be an option within U.S. Ob/Gyn residencies is underway [3]. At least 1 U.S. program is already incorporating this practice into their training [4]. Supporters of “tracking” believe that the typical 18 months throughout a typical 48-month residency program spent focused on gynecologic surgery is too little for comprehensive training [2]. Surgical training and resident exposure are of utmost importance as data continually demonstrates that volume matters; surgical morbidity is reduced when gynecologic surgery is performed by high volume surgeons [5–7]. Subspecialty training may be the only option to counteract this perceived deficiency in our traditional training model.

Less than half of first year fellows in 4 different subspecialties within Ob/Gyn (FPMRS, Onc, REI, and MFM) were considered prepared to function independently by their fellowship directors [8]. Active and former minimally invasive surgery fellows state one main reason for pursuing fellowship after completing a general surgery residency is to improve their surgical skills [9]. However, little is known about senior Ob/Gyn residents’ own perceptions of their preparedness to perform MIGS. Even less is known about the readiness of senior Ob/Gyn residents to perform both basic and *complex* MIGS. Recently the ACGME increased the minimum graduating requirement for minimally invasive hysterectomies from 35 to 70 [10]. With the calls to increase utilization of minimally invasive surgery undoubtedly more and more complex gynecologic cases will be attempted laparoscopically or vaginally [11, 12]. However, are we asking too much of undertrained and underprepared gynecologic surgeons? The aim of this study is to evaluate the confidence of senior U.S. Ob/Gyn residents in performing MIGS. Furthermore, we stratified this data by their intentions to pursue a surgical subspecialty fellowship (ONC, MIGS, FPMRS, REI, and PAGS) to determine if residents pursuing fellowship had differing levels of comfort with MIGS.

## Methods

We created and administered a voluntary survey for current U.S. Ob/Gyn residents using the Research Electronic Data Capture (REDCap) secure online platform. All study data were collected and managed using the REDCap tools hosted by The George Washington University [13, 14]. Because participation in this study was voluntary and information was kept de-identified, this study was determined to be exempt by the Institutional Review Board (IRB# 180767). Current U.S. accredited Ob/Gyn residency programs were identified using the ACGME online data system [15]. Program coordinators as well as program directors were contacted via email using publicly available information through the ACGME website. Throughout the months of February and March of 2019 program coordinators as well as program directors for all 280 registered Ob/Gyn residency programs (representing 5598 active Ob/Gyn residents) received a cover letter email including an electronic link to the survey and were asked to forward this link along to their residents. This email request was sent on three separate occasions over this two-month period in an effort to maximize the response rate. Resident responses to the survey were no longer accepted after June 1, 2019, which allowed a full 3 months for all interested residents to participate.

Because a validated survey assessing senior Ob/Gyn resident confidence in performing MIGS does not exist, all of the listed authors took part in creating a novel 58-item survey (Supplemental Figure 2). These authors included two fellows in MIGS, three MIGS fellowship trained high-volume surgeons from a single center, and one statistician. The authors did not employ any specific methodology to create the survey and questions were created and approved based on overall consensus. Senior Ob/Gyn residents were defined as residents in either the third or fourth year of residency. The survey contained basic demographic questions as well as questions designed to assess resident confidence in specific gynecologic procedures using a Likert scale from 1 to 5 (1 = Strongly Agree, 5 = Strongly Disagree). Additionally, some procedural questions were repeated with differing levels of complexity. For instance, confidence in laparoscopic hysterectomy was queried for a uterus less than 12-weeks size, greater than 12-week size but below the umbilicus, and for a uterus above the umbilicus. Similar question trees were designed to assess confidence in vaginal hysterectomy, laparoscopic myomectomy, hysteroscopic myomectomy, excision of endometriosis, specimen containment, tissue morcellation, and navigating the robotic surgical console. Residents were able to initiate the survey and finish at a later time, however, other than for demographic questions surveys could not be submitted with missing answer fields. If surveys returned had missing answer fields these fields were censored in the analysis. Chi-square or Fisher’s exact tests were used to examine associations between categorical variables, and 2-tailed between-groups t-tests were used to examine differences in means across groups. We used SAS 9.4 (SAS Institute, Cary, NC) for all analyses and a *P*-value < 0.05 was considered statistically significant.

## Results

We received 328 survey responses, 159 (48.5%) of which were from senior Ob/Gyn residents and included in the final analysis. Assuming all 5598 Ob/Gyn residents were given the opportunity to participate this yields an approximate 6% response rate for senior Ob/Gyn residents (159/2799). Basic demographic information as well as details regarding respondents residency programs are outlined in Table 1. Overall 76.2% of respondents felt that their residency program has adequately prepared them to perform MIGS. Residents who train with graduates of a fellowship in MIGS, regardless of whether or not the institution has a MIGS fellowship program, felt significantly more prepared to perform MIGS compared to residents without this exposure in their training (*p* = 0.02). Training environments including academic institutions, community programs, and academic-affiliated community programs did not have a significant impact on resident preparedness for MIGS (*p* = 0.44). There were no other statistically significant background differences found between residents who feel prepared to perform minimally invasive surgery and those that do not (Table 2).

**Table 1:** Demographic and background variables

Variable	Senior Residents (***N*** = 159)
Year in Residency	
Third	74 (46.5)
Fourth	85 (53.5)
Medical Degree	
Allopathic	140 (88.1)
Osteopathic	19 (11.9)
Gender	
Female	130 (81.8)
Male	28 (17.6)
Race	
White/Caucasian	105 (66.5)
Black or African-American	13 (8.2)
Asian	25 (15.8)
From Multiple Races	2 (1.3)
Age Between 26 and 30	90 (56.6)
Residency Training Type	
Academic	96 (60.4)
Community	17 (10.7)
Community-Academic Affiliated	46 (28.9)
Geographic Region of Training	
North East	75 (47.5)
North West	14 (8.9)
South East	49 (31.0)
South West	20 (12.6)
Feel Prepared to Perform MIGS	114 (76.19)
Applying for, or Accepted in, a Surgical Fellowship^a^	
Yes	40 (25.2)
No	108 (67.9)
Work in an Institution with Graduate(s) of a MIGS Fellowship	
Yes	93 (58.5)
No	64 (40.3)
Feel Prepared to Perform MIGS	114 (76.19)

Values reported as n (%)

Minimally Invasive Gynecologic Surgery (MIGS)

^a^ Surgical fellowships included MIGS, Female Pelvic Medicine and Reconstructive Surgery, Gynecologic Oncology, Reproductive Endocrinology and Infertility, and Pediatric and Adolescent Gynecology

**Table 2:** Characteristics of senior residents who feel their residency training has adequately prepared them to perform Minimally Invasive Gynecologic Surgery (*N* = 159)

Variable	Mean Likert Score	***P***-Value
Year in Residency		0.36
Third	2.21 ± 0.85	
Fourth	2.08 ± 0.91	
Medical Degree		0.33
Allopathic	2.16 ± 0.90	
Osteopathic	1.94 ± 0.75	
Race		
White/Caucasian	2.05 ± 0.91	0.10
Black/African-American	2.00 ± 0.63	0.60
Asian	2.43 ± 0.73	0.08
Gender		0.98
Female	2.14 ± 0.86	
Male	2.12 ± 0.99	
Age		0.27
26–30	2.07 ± 0.90	
31–35	2.20 ± 0.77	
> 35	3.00 ± 2.83	
Type of Residency Program		0.44
Academic	2.16 ± 0.94	
Community	1.88 ± 0.89	
Community-Academic Affiliated	2.20 ± 0.75	
Work with Graduate(s) of MIGS Fellowship		**0.02**
Yes	2.00 ± 0.81	
No	2.39 ± 0.94	

Values reported as mean ± SD on a 5-point Likert Scale (1 = Strongly Agree and 5 = Strongly Disagree)

*P* < 0.05 Bold

**Fig. 1:**
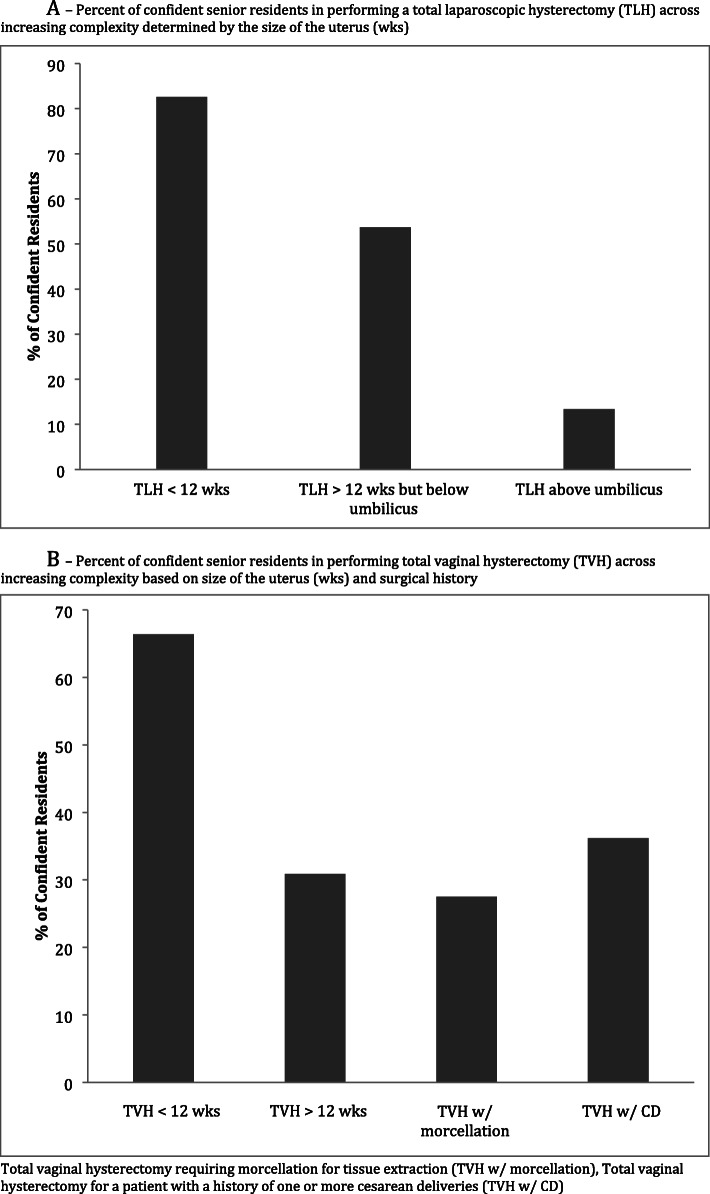
**a** Trends in Ob/Gyn senior resident confidence in total laparoscopic hysterectomy as surgical complexity increases. **b** Trends in Ob/Gyn senior resident confidence in total vaginal hysterectomy as surgical complexity increases

We did identify certain trends across procedures with respondents feeling less comfortable performing specific gynecologic procedures as the surgical complexity increased (Figs. 1a and b). Eighty-two percent of respondents felt confident in their ability to perform a total laparoscopic hysterectomy on a uterus less than 12-week size, however, this percent decreases to 13.4% when the uterus is above the umbilicus. Only 18% of respondents felt comfortable performing a laparoscopic myomectomy and this rate decreased to 13% if the procedure required more than one hysterotomy. Although the purpose of this study was not to assess junior residents (Ob/Gyn residents in either their first or second year) it is interesting to note the validation of our novel survey. Only 17.2% of junior residents felt confident in their ability to perform a total laparoscopic hysterectomy on a uterus less than 12-week size (compared to 82% for senior residents), and this percent drops to 5% when the uterus is above the umbilicus. Only 5.7% of junior residents felt comfortable performing a laparoscopic myomectomy and this rate decreased to 4.1% if more than one hysterotomy was required.

Using respondents mean Likert scale scores we identified the 5 procedures residents are least confident in performing as: 1) vaginal hysterectomy requiring vaginal morcellation for tissue extraction; 2) laparoscopic hysterectomy when the uterus is above the umbilicus; 3) excision of stage 3 or 4 endometriosis; 4) laparoscopic myomectomy requiring suturing; and 5) laparoscopic myomectomy requiring suturing of more than one hysterotomy. We found that the respondents who felt most confident (top 35th percentile) in performing these 5 most challenging procedures were significantly more likely to identify as male (*p* = 0.04). There is a non-significant trend towards higher confidence in the most challenging procedures among residents exposed to fellowship trained MIGS faculty (*p* = 0.08). No other statistically significant demographic or educational differences were identified between respondents who felt the most confident in performing the most challenging procedures and those who felt less confident (Table 3).

**Table 3:** Characteristics of the residents most confident in their ability to perform the most challenging minimally invasive surgeries

Variable	Most Confident Residents (***N*** = 56)	Less Confident Residents (***N*** = 103)	***P***-Value
Year in Residency			0.32
Third	23 (31.1)	51 (68.9)	
Fourth	33 (38.8)	52 (61.2)	
Medical Degree			0.61
Allopathic	48 (34.3)	92 (65.7)	
Osteopathic	8 (42.1)	11 (57.9)	
Race			0.60
White/Caucasian	39 (37.1)	66 (62.9)	
Black/African-American	7 (53.9)	6 (46.1)	
Asian	6 (24.0)	19 (76.0)	
Gender			**0.04**
Female	41 (31.5)	89 (68.5)	
Male	15 (53.6)	13 (46.4)	
Type of Residency Program			0.44
Academic	31 (32.3)	65 (67.7)	
Community	8 (47.1)	9 (52.9)	
Community-Academic Affiliated	17 (37.0)	29 (63.0)	
Work with Graduate(s) of MIGS Fellowship			0.08
Yes	29 (31.2)	64 (68.8)	
No	25 (39.1)	39 (60.9)	
Intend to Pursue, or Matched in, a Surgical Fellowship			0.09
Yes	16 (40.0)	24 (60.0)	
No	40 (37.0)	68 (63.0)	

Values reported as n (%). Not all variables represented; not all cumulative *n* = 159

*P* < 0.05 bold

We identified certain significant differences between residents who responded that they were either interested in, or had matched into, any one of the five surgical subspecialties of Ob/Gyn compared to residents that were not. Residents interested in surgical fellowships were more likely to be male (*p* = 0.005), train in an institution with both a division of MIGS (*p* = 0.02) and a MIGS fellowship program (*p* = 0.001), as well as train in an institution with a fellowship in FPMRS (*p* = 0.004). Overall, only 52.7% of respondents consider MIGS a subspecialty of Ob/Gyn in the same way they consider other accredited surgical subspecialties of Ob/Gyn (ONC, FPMRS, and REI). Less than half of the respondents not pursuing a fellowship (47.3%) plan on referring their complex gynecologic surgeries to a fellowship trained MIGS surgeon after completing their training. Resident confidence level in the most challenging surgical procedures had a trend-level association with their intentions to refer these procedures to a fellowship trained MIGS surgeon (*p* = 0.06).

## Discussion

We found that the majority of senior U.S Ob/Gyn residents who responded to this survey feel their training has prepared them to perform minimally invasive surgery. However, surgical confidence in specific procedures decreased when surgical complexity increased. Those residents exposed to fellowship trained MIGS surgeons appear to feel more prepared for minimally invasive surgery compared to residents lacking this exposure. One possible explanation for this is that residents in programs with MIGS fellowships complete a higher percentage of their cases, both simple and complex, through minimally invasive routes; thus the resident is exposed and has more confidence with these procedures.

With growing interest in the MIGS fellowship in MIGS and yearly increases in fellowship programs across the country, this should serve to increase resident comfort with MIGS [16]. In a similar study of 4th year U.S. Ob/Gyn residents, the data show that exposure to laparoscopic simulation (box trainers), as well as didactic lectures and a formal skills assessment were associated with perceived laparoscopic competence [17]. The impact of exposure to fellowship trained MIGS faculty was not assessed in that study. However, a different study by the same author did find that establishment of a MIGS fellowship at a single academic institution had a positive overall effect on resident experience and attitude without compromising surgical numbers [18].

A significantly larger proportion of respondents who were more confident in performing the 5 most challenging procedures identified as male. This finding is consistent with previously published data showing male residents were significantly more confident in performing total hysterectomy and treating endometriosis [17]. This finding likely reflects the disparity that exists between male and female surgical training. Previous study has found that male surgical trainees are given more autonomy in the operating room compared to their female counterparts [19]. No other background or educational variables appear to impact resident preparedness for complex minimally invasive surgery. There was no significant difference in surgical confidence between residents pursuing any of the surgical fellowships in Ob/Gyn and those residents planning on entering into general practice. Previous studies have found that less than half of fellowship program directors feel that their incoming fellows are “prepared” for fellowship [8, 20]. Specifically, fellowship program directors do not feel their fellows enter their subspecialty of training with adequate procedural skills to function independently [8, 20]. However, fellows own perceptions differ from their program directors; with a majority of fellows feeling their residency did prepare them for fellowship [21]. Data from General Surgery has shown that one of the main reasons residents pursue a fellowship in minimally invasive surgery is to increase their laparoscopic skills [9]. However, this study did not find that residents pursuing a fellowship in MIGS were any more or less confident in their surgical skills compared to those residents pursuing any other surgical fellowship or those residents not pursuing a fellowship.

Despite a majority of respondents own perceived confidence in MIGS there appears to be an inconsistency regarding complex gynecologic procedures. Fewer than one in five respondents feel confident in their ability to perform a laparoscopic myomectomy, a total laparoscopic hysterectomy for a uterus above the umbilicus, and a laparoscopic excision of advanced stage endometriosis. More concerning is the lack of intent to refer these complex cases to a fellowship trained MIGS surgeon if available. One possible, albeit worrying, explanation for this may be that these residents do not realize that these complex cases can be safely completed laparoscopically [22, 23]. Currently, no required case minimums exist for laparoscopic excision of endometriosis or laparoscopic myomectomy [10]. Residents are now required to perform a minimum of 70 minimally invasive hysterectomies, however, no system exists to account for the complexity of these minimally invasive cases [10]. Future study should aim to identify the mode of surgery and associated outcomes of these complex surgical patients not being referred to MIGS surgeons by recently graduated Ob/Gyns.

This study is not without its limitations. Unfortunately, there was a low resident participation rate and outreach was limited, as U.S. Ob/Gyn residents cannot be contacted directly through any publicly available database. According to the ACGME website during this study period there were 5598 active duty Ob/Gyn residents. However, we do not know how many of the 5598 were senior residents and there is also unfortunately no way to track how many senior Ob/Gyn residents were sent the survey link by their program. This also creates potential bias as we cannot assume the reasons certain program coordinators or directors would not forward this survey on to their senior residents. This low participation rate limits the generalizability of these results and likely results in this study being underpowered to identify potential significant differences. There is also an unavoidable selection bias inherent in the nature of this study. We relied on a 58-item survey that all authors had a part in creating as there is no validated survey to assess resident confidence in performing MIGS. Confidence in surgical procedures is also very subjective and does not necessarily equate to ability in performing the procedure. Strengths of this study come from the diversity of participants in terms of identified sex, geographic location, and residency type.

## Conclusions

To our knowledge this is also the first study to assess confidence in a variety of surgical procedures with question trees to simulate increasing surgical complexity. We also highlight 5 procedures residents are the least confident in performing and assess other indirect surgical skills underrepresented in the available literature. This data should serve as a guide for residency program directors, fellowship directors, and the ACGME, highlighting residents own perceived surgical deficiencies. Our findings stress the issue of whether case minimum numbers should be considered for certain complex procedures such as laparoscopic myomectomy and excision of endometriosis. Additionally, experience level should be taken into consideration when surgical credentialing privileges are being granted by institutions after graduation. Further study should target how to improve resident exposure and confidence in the procedures felt to be the most challenging.

## Supplementary information

**Additional file 1: Supplemental Figure S2.** Resident survey.

## Data Availability

All materials utilized in this study as well as available datasets can be produced and are available with reasonable request.

## Abbreviations

MIGS: Minimally Invasive Gynecologic Surgery
Ob/Gyn: Obstetrics and Gynecology
FPMRS: Female Pelvic Medicine and Reconstructive Surgery
Onc: Gynecologic Oncology
MFM: Maternal Fetal Medicine
REI: Reproductive Endocrinology & Infertility
PAGS: Pediatric and Adolescent Gynecology
ACGME: Accreditation Council for Graduate Medical Education
