# Supplementary material for: Ob/Gyn resident self-perceived preparedness for minimally invasive surgery
Source: BMC Med Educ. 2020 Jun 5;20:185. doi: 10.1186/s12909-020-02090-9 (PMC7275515; doi:10.1186/s12909-020-02090-9)
Supplement: Supplementary file 1 — Additional file 1: Supplemental Figure S2. Resident survey. [file 12909_2020_2090_MOESM1_ESM.pdf]

Resident Preparedness for Minimally Invasive Surgery

Please complete the survey below. If you are interested in being rewarded for your participation in this survey please enter your preferred email when prompted. This email will be kept confidential and is only being used for communication for those participants randomly selected for reward.

Thank you!

Please enter your preferred email address so that you may be contacted in the event you win one of the visa gift cards for your participation in this survey

____
(This information will be kept confidential and will only be visible to the principal investigator (Klebanoff J). The purpose for requiring a personal email address is to ensure that participants are only completing one survey and to award the random winners of the visa gift cards.)

Demographic Information

I am currently a _____ year OB/Gyn resident (choose one)

- ○ First
- ○ Second
- ○ Third
- ○ Fourth

In what geographic region is your residency located?

- ○ North East
- ○ North West
- ○ South East
- ○ South West

I graduated from an _____ medical school

- ○ Osteopathic (DO)
- ○ Allopathic (MD)

I attend a residency within the United States

- ○ Yes
- ○ No

I identify as _____

- ○ Female
- ○ Male
- ○ Other

How would you describe yourself (choose all that apply)

- ☐ White
- ☐ Black or African American
- ☐ Asian
- ☐ American Indian or Alaska Native
- ☐ Native Hawaiian or other Pacific Islander
- ☐ From multiple races
- ☐ Hispanic or Latino
- ☐ Other

My age is

- ○ < 20
- ○ 20-25
- ○ 26-30
- ○ 31-35
- ○ > 35

My residency program is considered a(n) _____ program

- ○ Academic-University based
- ○ Community
- ○ Community-Academic affiliated

What percentage of the residents from your training program go on to pursue a fellowship (Minimally Invasive Gynecologic Surgery, Gynecologic Oncology, Maternal Fetal Medicine, Female Pelvic Medicine and Reconstructive Surgery, Family Planning, Pediatric and Adolescent Gynecology, or Reproductive Endocrinology and Infertility)?

- ○ < 25%
- ○ 25-50%
- ○ 51-75%
- ○ > 75%
- ○ Unsure

Do you work in an institution with graduates of a fellowship in Minimally Invasive Gynecologic Surgery (MIGS)?

- ○ Yes
- ○ No
- ○ Unsure

Which of the following divisions within the department of Ob/Gyn does your institution have (choose all that apply)?

- ☐ Minimally Invasive Gynecologic Surgery (MIGS)
- ☐ Gynecologic Oncology (Gyn Onc)
- ☐ Female Pelvic Medicine and Reconstructive Surgery (FPMRS)
- ☐ Reproductive Endocrinology and Infertility (REI)
- ☐ Pediatric and Adolescent Gynecology (PAGS)
- ☐ Unsure
- ☐ None

Which of the following fellowships does your institution offer (choose all that apply)?

- ☐ Minimally Invasive Gynecologic Surgery (MIGS)
- ☐ Gynecologic Oncology (Gyn Onc)
- ☐ Female Pelvic Medicine and Reconstructive Surgery (FPMRS)
- ☐ Reproductive Endocrinology and Infertility (REI)
- ☐ Pediatric and Adolescent Gynecology (PAGS)
- ☐ Unsure
- ☐ None

Are you applying for, or have you been accepted to, a SURGICAL fellowship in Ob/Gyn (MIGS, Gyn Onc, FPMRS, REI, or PAGS)?

- ◯ Yes
- ◯ No
- ◯ Yes, but I did not match
- ◯ Undecided

In which of the fellowships did you apply but not match?

- ◯ Minimally Invasive Gynecologic Surgery (MIGS)
- ◯ Gynecologic Oncology (Gyn Onc)
- ◯ Female Pelvic Medicine and Reconstructive Surgery (FPMRS)
- ◯ Reproductive Endocrinology and Infertility (REI)
- ◯ Pediatric and Adolescent Gynecology (PAGS)

Do you plan on reapplying at any time in the future?

- ◯ Yes
- ◯ No
- ◯ Unsure

In which surgical fellowship have you applied, or matched?

- ◯ Minimally Invasive Gynecologic Surgery (MIGS)
- ◯ Gynecologic Oncology (Gyn Onc)
- ◯ Female Pelvic Medicine and Reconstructive Surgery (FPMRS)
- ◯ Reproductive Endocrinology and Infertility (REI)
- ◯ Pediatric and Adolescent Gynecology (PAGS)

How many surgical fellowship programs did you apply to?

- ◯ < 10
- ◯ 10-20
- ◯ 20-30
- ◯ > 30

During residency I feel that my surgical skills are _____ to my co-residents

- ◯ Superior
- ◯ Equal
- ◯ Inferior
- ◯ Unsure

General Questions for All Residents

The following questions are to be answered by ALL residents

	Strongly Agree	Agree	Neutral	Disagree	Strongly Disagree	Not Applicable
I feel my residency training adequately prepared me to be a competent minimally invasive surgeon	○	○	○	○	○	○
I feel confident in my ability to perform a total laparoscopic hysterectomy on a uterus less than 12 week size (as the primary surgeon)	○	○	○	○	○	○
I feel confident in my ability to perform a total laparoscopic hysterectomy on a uterus greater than 12 week size but below the umbilicus (as the primary surgeon)	○	○	○	○	○	○
I feel confident in my ability to perform a total laparoscopic hysterectomy on a uterus above the umbilicus (as the primary surgeon)	○	○	○	○	○	○
I feel confident in my ability to perform a vaginal hysterectomy on a uterus less then 12 week size (as the primary surgeon)	○	○	○	○	○	○
I feel confident in my ability to perform a vaginal hysterectomy on a uterus greater than 12 weeks size (as the primary surgeon)	○	○	○	○	○	○
I feel confident in my ability to perform a vaginal hysterectomy on a fibroid uterus that will require vaginal morcellation for tissue extraction (as the primary surgeon)	○	○	○	○	○	○
I feel confident in my ability to perform a vaginal hysterectomy in a patient with a history of 1 or more cesarean sections (as the primary surgeon)	○	○	○	○	○	○

I feel confident in my ability to perform a laparoscopic myomectomy requiring laparoscopic suturing (as the primary surgeon)	○	○	○	○	○	○
I feel confident in my ability to perform a laparoscopic myomectomy requiring laparoscopic suturing of multiple hysterotomies (as the primary surgeon)	○	○	○	○	○	○
I feel confident in my ability to perform an excisional procedure for stage 3 or 4 endometriosis (as the primary surgeon)	○	○	○	○	○	○
I feel confident in my ability to laparoscopically navigate the retroperitoneum to identify landmark anatomy	○	○	○	○	○	○
I feel confident in my ability to perform major laparoscopic surgery using the robotic platform	○	○	○	○	○	○
I feel confident in my ability to align and dock the robotic platform in preparation for surgery	○	○	○	○	○	○
I feel confident in my ability to troubleshoot the robotic platform when issues/errors were encountered before or during a case	○	○	○	○	○	○
I feel confident in my ability to bag/contain a specimen less than 10 cm with minimal assistance	○	○	○	○	○	○
I feel confident in my ability to bag/contain a specimen between 10 and 15 cm with minimal assistance	○	○	○	○	○	○
I feel confident in my ability to bag/contain a specimen larger than 15 cm with minimal assistance	○	○	○	○	○	○

I feel confident in my ability to perform both vaginal and abdominal manual contained morcellation	○	○	○	○	○	○
I feel confident in my ability to perform office hysteroscopy in an awake patient with either local or no anesthesia	○	○	○	○	○	○
I feel confident in my ability to perform a hysteroscopic myomectomy for Type 0 fibroid(s)	○	○	○	○	○	○
I feel confident in my ability to perform a hysteroscopic myomectomy for Type 1 fibroid(s)	○	○	○	○	○	○
I feel confident in my ability to perform a hysteroscopic myomectomy for Type 2 fibroid(s)	○	○	○	○	○	○

Questions Specific for Residents Interested in MIGS

The following seven (7) questions are ONLY for residents who have applied, plan to apply, or have matched into a fellowship in MIGS. If this does not describe you please select 'NOT APPLICABLE' for each question.

	Strongly Agree	Agree	Neutral	Disagree	Strongly Disagree	Not Applicable
I chose to pursue a MIGS fellowship because I am hoping not to practice OB	○	○	○	○	○	○
I considered applying to other surgical subspecialties (Gyn Onc, UroGyn, REI) before ultimately deciding to pursue a fellowship in MIGS	○	○	○	○	○	○
Part of my decision to apply for a fellowship in MIGS was to teach residents and/or fellows minimally invasive surgery techniques	○	○	○	○	○	○
I sought, or am seeking, a fellowship program with increased exposure to the management of chronic pelvic pain in women	○	○	○	○	○	○
I sought, or am seeking, a fellowship program with increased exposure to the management pelvic organ prolapse and/or urinary incontinence	○	○	○	○	○	○
After fellowship I am hoping to work in an academic institution	○	○	○	○	○	○
After fellowship I am hoping to work in an institution with a division of minimally invasive gynecologic surgery	○	○	○	○	○	○

Questions Specific to Residents Not Interested in MIGS

The following seven (7) questions are ONLY for residents NOT interested in pursuing a fellowship in MIGS. If this does not describe you please select 'NOT APPLICABLE' for each question.

	Strongly Agree	Agree	Neutral	Disagree	Strongly Disagree	Not Applicable
Following completion of my residency I hope part of my employment allows me to teach residents minimally invasive surgery techniques	○	○	○	○	○	○
I am confident in my ability to both medically and surgically manage chronic pelvic pain in women	○	○	○	○	○	○
I feel confident in my ability to manage pelvic organ prolapse and/or urinary incontinence using both surgical and non-surgical techniques	○	○	○	○	○	○
I am hoping to work in an academic institution	○	○	○	○	○	○
I am hoping to work in an institution with a division of minimally invasive gynecologic surgery	○	○	○	○	○	○
In my practice I plan on referring complex gynecologic cases to a fellowship trained minimally invasive gynecologic surgeon whenever available	○	○	○	○	○	○
I consider MIGS a subspecialty of Obstetrics and Gynecology in the same way that Gynecologic Oncology, FPMRS, and REI are subspecialties	○	○	○	○	○	○
